# Skin-Limited Graft-versus-Host Disease after Pancreatic Transplantation

**DOI:** 10.1155/2017/4823870

**Published:** 2017-07-18

**Authors:** Muneeb Ilyas, Elika Hoss, David J. DiCaudo, Hasan Khamash, Mark R. Pittelkow, Amit Sharma

**Affiliations:** ^1^Department of Dermatology, Mayo Clinic, Scottsdale, AZ, USA; ^2^Department of Nephrology, Mayo Clinic, Phoenix, AZ, USA

## Abstract

**Introduction:**

The phenomenon of graft-versus-host disease, a solid organ transplant recipient, is a rare development with a very poor prognosis.

**Case Presentation:**

A 40-year-old woman with type 1 diabetes developed cutaneous graft-versus-host disease following second pancreas transplantation.

**Conclusion:**

The development of a nonspecific rash in the early posttransplant period following a pancreas transplant warrants suspicion for graft-versus-host disease.

## 1. Introduction

Graft-versus-host disease (GVHD) is a common complication after bone marrow transplantation; GVHD, however, is rare after solid organ transplant (SOT), where it carries a very poor prognosis. We report a unique case of skin-limited GVHD after pancreatic transplantation.

## 2. Report of Case

**Figure 1:**
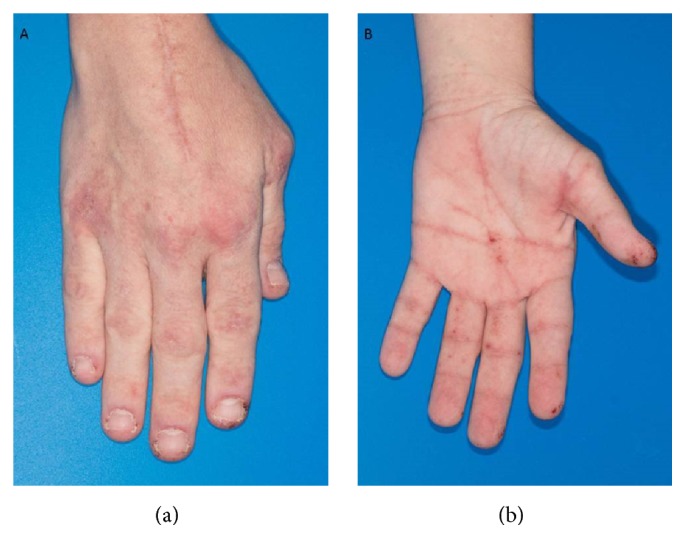
(a) Right dorsal hand with scaly pink plaques. (b) Left palmar surface with punctate firm hemorrhagic papules.

A 40-year-old woman with trisomy 21 developed an acute eruption on the hands, legs, and face 57 days after her second pancreas transplant for type 1 diabetes. Initial pancreas transplant was 12 years prior, complicated by antibody-mediated rejection. Patient received her second pancreas from a deceased male donor. She had induction with thymoglobulin and oral corticosteroids. Three units of irradiated blood were given in the operating room. The patient had stable white blood cell counts following transplantation with no evidence of leukopenia. Two weeks prior to her eruption, she had elevated liver enzymes, which resolved after discontinuation of isoniazid, prescribed for latent tuberculosis, and fluconazole. On physical examination, there were scaly pink plaques in the periocular area, dorsum of the hands, and the lower legs. In addition, she had punctate firm hemorrhagic papules of the fingertips and palms (Figure 1). The patient also had mild cervical adenopathy. Two skin biopsies were performed on the different morphologies on the hands. Immunosuppression was not reduced or altered following the onset of the rash.

**Figure 2:**
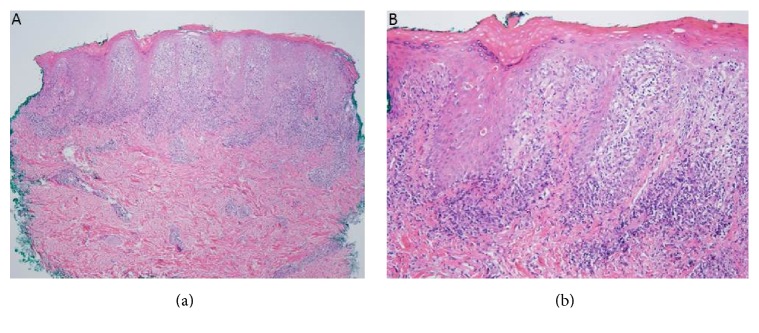
Hematoxylin-eosin-stained biopsy specimens from left 2nd digit. (a) Dense superficial to middermal lymphocytic infiltrate with few scattered eosinophils (40x magnification). (b) Scattered apoptotic keratinocytes within the epidermis and vacuolization at the dermal-epidermal junction (100x magnification).

Histopathologic evaluation of both specimens revealed scattered apoptotic keratinocytes within the epidermis, with vacuolization at the dermal-epidermal junction. There was a dense superficial to middermal lymphocytic infiltrate, with some scattered eosinophils (Figure 2). Additional finding of hemorrhage was seen in specimen from the fingertip. Findings were consistent with lichenoid tissue reaction and thus raised concern for lichenoid GVHD (grade 2) versus lichenoid drug eruption. Fluorescence in situ hybridization (FISH) testing for chromosomes X and Y found the lymphocytes as XY, while the surrounding normal tissue was XX, indicating that the inflammatory infiltrate was primarily of male (donor) origin, confirming the diagnosis of GVHD. Chimerism analysis on the peripheral blood showed 0% donor DNA.

Patient was diagnosed with cutaneous GVHD and was treated with topical corticosteroids. The rash and cervical adenopathy promptly resolved. Investigation for other organ involvement was done with routine laboratory analysis, as well as EGD and colonoscopy. No other organ involvement was identified, and she remained stable with no progression of her disease.

## 3. Discussion

To our knowledge, this is the first case of skin-limited GVHD after SOT. GVHD is a rare, but devastating complication of SOT. The incidence of GVHD after SOT is highest following small intestinal transplantation (5.6%) [1]. Following liver transplantation, GVHD is estimated to occur in less than 1% of cases and carries a poor prognosis with a mortality rate greater than 90% [2]. There have only been a handful of reports of GVHD after pancreatic transplantation [3].

Common clinical manifestations of acute GVHD following SOT include nonspecific rash, fever, vomiting, abdominal pain, and diarrhea. Histopathologic evaluation of skin affected by GVHD shows a lichenoid tissue reaction, but this is not pathognomonic. Further evaluation with FISH analysis and genetic chimerism analysis of peripheral blood confirms the diagnosis.

In summary, we present a case of skin-limited GVHD after pancreas transplantation. It is important to be suspicious for GVHD in SOT recipients presenting in the early posttransplant period for evaluation of rash. The authors recommend early skin biopsy in these patients. If there is histologic suspicion for GVHD, FISH analysis and peripheral blood chimerism analysis may be useful for confirmation. Concern for cutaneous GVHD should prompt further evaluation of other organs for GVHD. Though there is no consensus treatment for GVHD following SOT, tumor necrosis factor-*α* inhibitors and T-cell depleting antibodies are encouraging therapeutic options [4, 5].
